# LATENT CLASS ANALYSIS OF RE-EXPERIENCING VIOLENCE ACROSS THE LIFE COURSE

**DOI:** 10.1093/geroni/igz038.1271

**Published:** 2019-11-08

**Authors:** Jooyoung Kong, Scott Easton

**Affiliations:** 1 University of Wisconsin-Madison, Madison, United States; 2 Boston College School of Social Work, Chestnut Hill, Massachusetts, United States

## Abstract

Guided by the cumulative disadvantage hypothesis, the present study examines recurrent victimization experiences across the life course and their impact on psychological health in later life. Using data from the 2010-2011 Wisconsin Longitudinal Study, we explored the latent structure of histories of childhood maltreatment (i.e., neglect, emotional/physical abuse, witness of domestic violence) and elder abuse victimization among 5,968 older adults (average age of 71 years). We also investigated whether membership in specific latent classes, particularly experiencing both childhood and elder victimization, would be associated with psychological functioning in late life. We identified five latent classes: “Never victimized” (66% of respondents), “Abused as child” (16%), “Abused and neglected as child” (9%), “Abused as elder” (6%), and “Abused as child and elder” (2%). Also, the “abused as child and elder” class consistently was associated with negative psychological outcomes (i.e., distress and somatic symptom severity) and lower levels of psychological well-being.

